# Experimental and Molecular Docking Studies of Cyclic Diphenyl Phosphonates as DNA Gyrase Inhibitors for Fluoroquinolone-Resistant Pathogens

**DOI:** 10.3390/antibiotics11010053

**Published:** 2022-01-01

**Authors:** Neveen M. Saleh, Yasmine S. Moemen, Sara H. Mohamed, Ghady Fathy, Abdullah A. S. Ahmed, Ahmed A. Al-Ghamdi, Sami Ullah, Ibrahim El-Tantawy El Sayed

**Affiliations:** 1 Department of Microbiology, National Organization for Drug Control and Research, Giza 12553, Egypt; sara_hussein_moh@yahoo.com; 2 Clinical Pathology Department, National Liver Institute, Menoufia University, Shebin El-Kom 32511, Egypt; yasmine.moemen@gmail.com; 3 Chemistry Department, Faculty of Science, Menoufia University, Shebin El-Kom 32511, Egypt; ghadyfathy96@gmail.com (G.F.); chemist_abdullah_2009@yahoo.com (A.A.S.A.); 4 Department of Physics, Faculty of Science, King Abdulaziz University, Jeddah 21589, Saudi Arabia; agamdi@kau.edu.sa; 5 Research Center for Advanced Materials Science (RCAMS), King Khalid University, P.O. Box 9004, Abha 61413, Saudi Arabia; samiali@kku.edu.sa; 6 Department of Chemistry, College of Science, King Khalid University, P.O. Box 9004, Abha 61413, Saudi Arabia

**Keywords:** cyclic diphenylphosphonate, quinoline, antibacterial activity, DNA gyrase inhibitor, molecular docking, magnesium ion

## Abstract

DNA gyrase and topoisomerase IV are proven to be validated targets in the design of novel antibacterial drugs. In this study, we report the antibacterial evaluation and molecular docking studies of previously synthesized two series of cyclic diphenylphosphonates (**1a**–**e** and **2a**–**e**) as DNA gyrase inhibitors. The synthesized compounds were screened for their activity (antibacterial and DNA gyrase inhibition) against ciprofloxacin-resistant *E.coli* and *Klebsiella pneumoniae* clinical isolates having mutations (deletion and substitution) in QRDR region of DNA gyrase. The target compound (**2a**) that exhibited the most potent activity against ciprofloxacin Gram-negative clinical isolates was selected to screen its inhibitory activity against DNA gyrase displayed IC_50_ of 12.03 µM. In addition, a docking study was performed with inhibitor (**2a**), to illustrate its binding mode in the active site of DNA gyrase and the results were compatible with the observed inhibitory potency. Furthermore, the docking study revealed that the binding of inhibitor (**2a**) to DNA gyrase is mediated and modulated by divalent Mg^2+^ at good binding energy (–9.08 Kcal/mol). Moreover, structure-activity relationships (SARs) demonstrated that the combination of hydrazinyl moiety in conjunction with the cyclic diphenylphosphonate based scaffold resulted in an optimized molecule that inhibited the bacterial DNA gyrase by its detectable effect in vitro on gyrase-catalyzed DNA supercoiling activity.

## 1. Introduction

Infections are among the major causes of human morbidity and mortality. The pharmaceutical industry is unable to keep up with the growing need for effective novel antibacterial drugs [1]; The main reason for this situation is the rapid bacterial adaptation to antibiotics; which, results in resistance development after antibacterial drugs are introduced into clinical use. Antibiotic resistance (AR) has been deemed as one of the most threats to global public health by the World Health Organization [1]. In 2050, an estimated number of 10 million deaths per year will be attributed to AR, thus proper action needs to be applied to stop this negative development [2]. Around 50% of the antimicrobial drugs prescribed for human diseases, found to be unnecessary [3]. This use, misuse, overuse, or over-the-counter has driven the major source towards AR [4].

DNA gyrase and topoisomerase IV are essential bacterial enzymes that represent an important target for novel antibacterial drug development [5,6]. An important strategy in fighting antibiotic resistance is the discovery, development of novel antibiotics, and increasing the efficacy of the antibiotic that is already in a clinical study [7]. In this context, α-aminophosphonates gained great interest by medicinal chemists because of their diverse biological and industrial applications such as antibacterial [8,9,10,11,12], anticancer [13,14,15], enzyme inhibitors [16,17], and chelating material [18,19,20,21] which made them a promising drug candidate for further optimization. These compounds are phosphorus analogs of naturally occurring α-amino acids and therefore, are considered promising in the field of drug discovery and development [22]. Moreover, quinoline-containing heterocyclic compounds received much attention due to their wide pharmacological applications [23,24,25]. The hybridization of quinoline moiety with α-aminophsophonate is expected to have a noticeable synergistic effect on biological activity. The present work has been carried out as part of our ongoing program for developing novel antibacterial agents which are based on α-aminophsophonates. In our previous work, we have reported a new class of cyclic α-aminophophonates bearing quinoline and hydrazine moiety with potent antibacterial activity [26]. These results prompted us to further extend and evaluate their antibacterial activity to understand the mode of action of the active compounds, for example, the determination of DNA gyrase binding affinity as potential inhibitors. In addition, a molecular docking approach will be applied to select the most promising inhibitor(s) for further lead optimization. Moreover, the structure-activity relationships (SARs) will be investigated to identify the most potent inhibitor that can be used as a template to further design novel compounds targeting DNA gyrase and effective against resistant bacterial strains such as fluoroquinolone-resistant pathogens. Herein, we describe the discovery of bacterial DNA gyrase inhibitors via binding to, and stabilization of, DNA cleavage complexes. 

## 2. Results and Discussion

### 2.1. Chemistry

**Figure 1:**
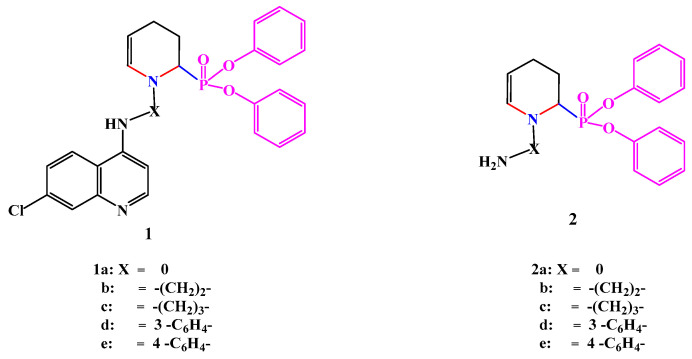
Structures of cyclic diphenylphosphonates **1** and **2**.

A two series of cyclic diphenylphosphonates (**1a**–**e** and **2a**–**e**) were prepared by following the procedure previously reported by us starting from glutaraldehyde, amines, diphenylphosphite, and Lewis acid catalyst. The structures of the synthesized compounds were confirmed based on their spectral data and in good agreement with the proposed structures and with those reported in the literature [26]. The two synthesized series of compounds used for current biological screening are listed in the following Figure 1.

### 2.2. In Vitro Antibacterial Activity

**Figure 2:**
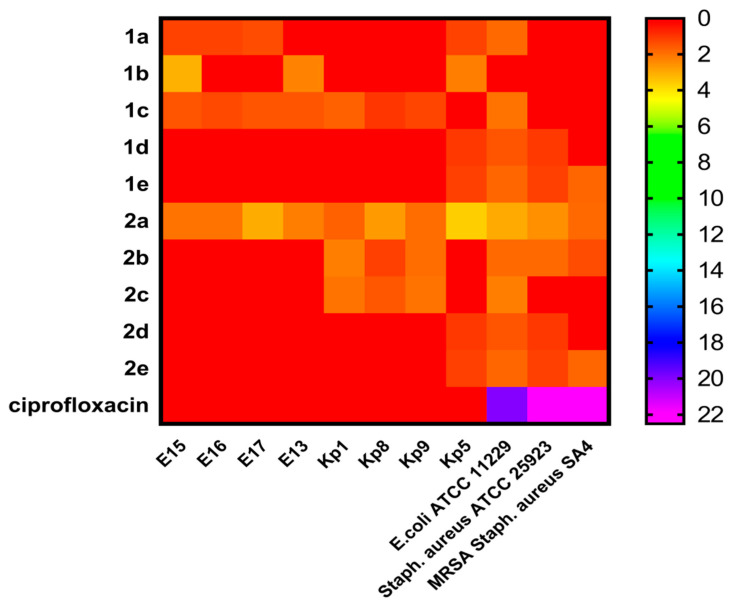
Heatmap Profile showed the sensitivity of tested MDR resistant Gram-positive and Gram-negative bacteria toward the tested two groups synthetic chemical compounds (**1a**–**e**, and **2a**–**e**) at a concentration (100 mg/mL) using agar well diffusion method. Scale from low inhibition zone diameter to high inhibition zone diameter. Red color represents low scale to violet color that represents a high scale.

Recently, two series of synthetic chemical compounds (**1a**–**e** and **2a**–**e**) were tested against different highly virulent strains of clinical isolates *E.coli*, *K. pneumonia*, and *Staphylococcus aureus* MRSA that previously showed resistance to ciprofloxacin [25,27,28,29], besides two reference strains (*Staphylococcus aureus* ATCC 25923, and *E. coli* ATCC 11229) and exhibited good antibacterial activity [26] as depicted in Figure 2 and Table S1 (cf. supplementary file). 

The results of in vitro antibacterial activity from the heatmap (Figure 2, Table S1) and the structure-activity relationships study (SARs), revealed that the first series (**1a**–**e**) bearing quinoline moiety, compound **1c** with a 3-carbon spacer between the two nitrogen showed activity against almost all tested Gram-negative bacteria. Moreover, compound (**1a**) without spacer has potent activity against three pathogens of *E. coli* and one pathogen of *K. pneumonia*. On the other hand, compound (**1b**) with two carbon spacers has antibacterial activity against only two *E. coli* strains (E13 and E15), while compounds (**1d** and **1e**) with rigid phenyl ring spacer showed potent antibacterial activity against both Gram-positive and Gram-negative bacteria and compound (**1e**) showed activity against both sensitive and resistant strains. For the second synthesized series (**2a**–**e**), compound (**2a**) without quinoline ring and carbon spacer showed antibacterial activity against all tested Gram-positive and Gram-negative pathogens, followed by compounds (**2b** and **2c**) with flexible two and three carbon spacers, respectively, that have been shown activity against only three Gram-negative strains of *K. pneumonia*. 

However, compounds (**2d** and **2e**) with rigid phenyl ring spacer showed the same activity as (**1d** and **1e**) from the first series. In addition, the results revealed that *K. pneumonia* (Kp5) was the sensitive isolate to almost all tested compounds. A comparison between cyclic diphenylphosphonate with quinoline motif and the corresponding ones without revealed that the former with quinoline was the most potent against MDR resistant Gram-positive and Gram-negative bacteria. From SARs study it was concluded that the difference in antibacterial activity between the two series of compounds was a result of the different substitutions on the cyclohexene ring nitrogen attached to the chiral carbon-bearing the diphenylphosphonate structural motif.

### 2.3. DNA Gyrase Mutation in Ciprofloxacin-Resistant Clinical Isolates E. coli (E17) and K. pneumonia (Kp8)

Exploring the resistance mechanism that bacteria used to resist the aaction of the antibiotic to show if the resistance was due to mutation in amino acids codon in quinolone resistance determining region that shas been associated with DNA gyrase where the structural topoisomerase changes reducing the affinity of this enzyme to fluoroquinolones are caused by mutations in the quinolone resistance-determining regions (QRDR) of *gyrA*/*gyrB*/*parC* and/or *parE* genes that will help to figure out the way to discover a new treatment approach. 

**Figure 3:**
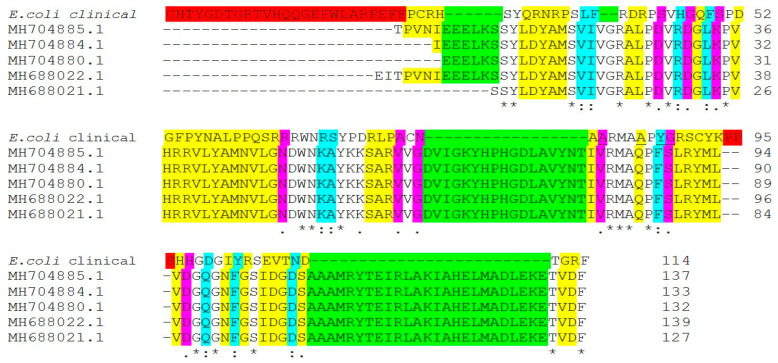
Amino Acid Multiple Sequence Alignment of *gyrA* (DNA gyrase subunit A) gene mutations in ciprofloxacin-resistant *Escherichia coli* clinical isolate (E17) compared with another *gyrA* gene in NCBI database. The alignment sequence was performed using Clustal W2 sequence alignment. Different colors indicates the difference in sequence aligment, red color indicates genes codon in our clinical isolte deleted in compared gene bank isolates, green colors indicates genes codon deleted in out tested isolates, “*” conserved sequences indicates amino acid have single, fully conserved residue (identical); “.” violet color Indicates conservation between groups of strongly similar properties; “:” blue color indicates non-conservative mutations that amino acid have different properties.

**Figure 4:**
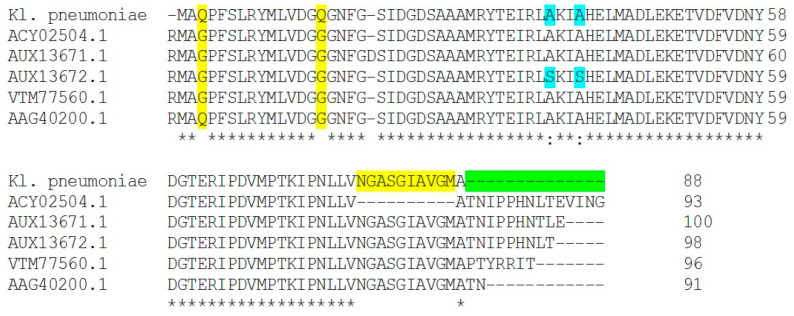
Amino Acid Multiple Sequence Alignment of *gyrA* (DNA gyrase subunit A) gene mutations in ciprofloxacin-resistant *Klebsiella pneumoniae* clinical isolate (Kp8) compared with another *gyrA* gene in NCBI database, mutation by deletion from position 75 to 88 of QRDR. The alignment sequence was performed using Clustal W2 sequence alignment. Different colors indicates the difference in sequence aligment, red color indicates genes codon in our clinical isolte deleted in compared gene bank isolates, green colors indicates genes codon deleted in out tested isolates, “*” conserved sequences indicates amino acid have single, fully conserved residue (identical); “:” blue color indicates non-conservative mutations that amino acid have different properties.

We studied that mechanism in both clinical isolates *E. coli* (E17) and *K. pneumonia* (Kp8). To identify substitution and deletion mutations, manually *QRDR* sequences were compared with GenBank sequences. Nucleotide sequences coding amino acid 70–88 in the gyrA QRDR were analyzed. The National Institutes of Health (NCBI) Web site was used to perform BLASTP analysis. To map the relative locations of *gyrA*, NCBI BLASTN and BLASTX, accessed through (https://blast.ncbi.nlm.nih.gov/Blast.cgi, accessed on 5 May 2020), were used to search the *E. coli* and *K. pneumonia* genome for *gyrA* gene, and the alignment sequence for mutation was performed using Clustal W2 sequence alignment (Figure 3 and Figure 4).

By comparing *gyrA* gene of ciprofloxacin-resistant *E.coli* (E17) clinical isolate with other *gyrA* genes in NCBI database (Figure 3), the mutation was noted at codon positions 85 glutamine (Gln)→alanine (Ala), 87 phenylalanine (Phe)→tyrosine (Tyr), and 88 serine (Ser)→glycine (Gly) of QRDR, which resulted in a substitution of Glutamine, Phenylalanine, and serine with alanine, tyrosine, and glycine, respectively. Mutations in *gyrA* were defined as one of the most common mechanisms of fluoroquinolone-resistance in different bacterial species [6,30], and usually detected at either codon 83 or 87, and/or the *parC* gene [31]. Mutations of *gyrA* at codons 67, 81, 82, 83, 84, 87, and 106 are responsible for the development of quinolone resistance in *E. coli*, while in clinical isolates, the most common mutation is at codon 87 [32]. Accumulation of Ser83 Leu and Asp87Asn mutations in the *gyrA* gene of *E. coli* was common (48). In addition to some unique mutations in codons 60, 64, 111 of *gyrA*, silent mutations at codons 85, 86, and 91 in resistant strains were observed [33]. Different changes of amino acid residues (codon Asp-87) between isolates having the same genotype observed, which suggested that the amino acid mutations at codon 87 (and possibly the development of high-level fluoroquinolone-resistance) might have occurred after the transmission/sharing of a precursor strain carrying the Ser-83→Leu mutation [34].

In our study, mutation by deletion from position 75 to 88 of QRDR was detected in Kp8 clinical isolate (Figure 4). Acquisition of mutations in *gyrA*, as well as *parC* genes, suggested playing a significant role in causing high quinolones-resistance levels in certain claustral lineages of *K. pneumoniae* [6]. Isolates carrying such mutations were found to have high MICs (˃16 μg/mL), which indicates that these alterations in *gyrA* are responsible for conferring high-level ciprofloxacin resistance [35]. Mutations at codons such as 83 and 87 in *gyrA* gene have been reported as the most common mutation points causing major alterations among clinical strains resulting in fluoroquinolone resistance [36].

Due to the useful characteristics of fluoroquinolone as potency, the spectrum of activity, oral bioavailability, and generally good safety profile, they were used extensively for multiple clinical indications throughout the world they still clinically valuable, but fluoroquinolone use has become limited in some clinical settings, as bacterial resistance has emerged over time, therefore, the screening of novel DNA gyrase inhibitor has been needed to solve this issue [37].

### 2.4. DNA Supercoiling Assay of Compound (***2a***) against DNA Gyrase Enzyme Activity

We rationalized the inhibitory effect of hydrazine linker exerts on the cell wall, as well as DNA gyrase inhibition. Reports stated that ciprofloxacin-resistance evolved in bacteria due to alterations in DNA gyrase/topoisomerase IV or due to a decrease in intracellular drug levels caused by changes in membrane permeability or overexpression of drug efflux pumps of the cell wall [6,38] and that what have been proved in our investigation, bacterial strains showed ciprofloxacin-resistance due to DNA alteration. To discover novel inhibitors that would act on microbial topoisomerases that are resistant to the known DNA gyrase inhibitors, we utilized synthetic chemical compound (**2a**) screening against gyrase of *E. coli* DNA as a model. 

According to the screening results of the antibacterial activity, compound (**2a**) was selected to use as a gyrase enzyme inhibitor of *E. coli* DNA gyrase, the compound markedly inhibited both ciprofloxacin-resistant mutant and the wild-type strain with different behavior toward Gram-positive bacteria that was less susceptible in comparison with Gram-negative bacteria. Importantly, this inhibition was performed through this mechanism depending on genetic approaches to probe the nature of the mechanism action.

However, we postulated that compound **2a** may inhibit DNA gyrase which is considered the primary target in Gram-negative bacteria as well as topoisomerase V by inhibiting both wild-type and CIP-resistant *Staphylococcus aureus MRSA*. Our data was supported by Doddaga et al., 2013 who proved the inhibitory effect of α-aminophosphonate against DNA Gyrase [39].

**Figure 5:**
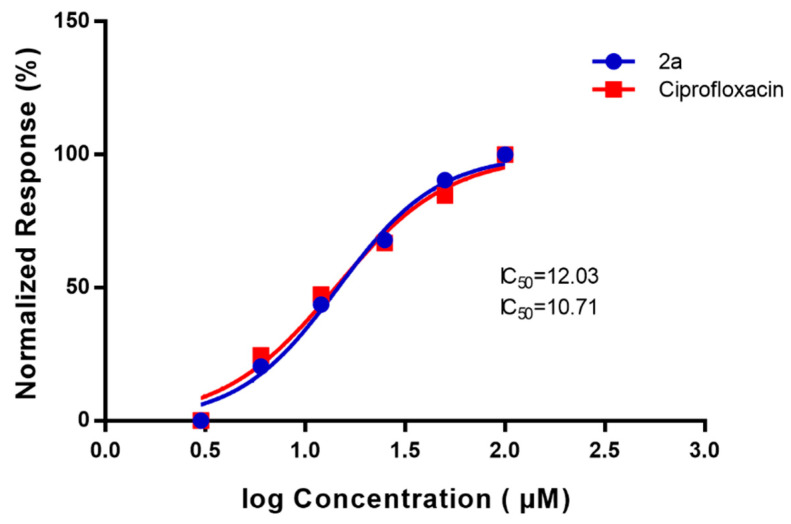
Dose-response inhibition curve for *E. coli* DNA gyrase-dependent supercoiling activity in the presence of cyclic diphenylphosphonate **2a**, ciprofloxacin with different concentrations of inhibitor (**2a**), and ciprofloxacin (standard) which showed 50% inhibition after 30 min as affecting DNA gyrase enzyme and the DNA was separated on a 1.0% (*w*/*v*) agarose gel. The fluorescence intensity of the supercoiled DNA band was normalized to the DMSO solvent control.

In our study, we tested the effect of compound (**2a**) on supercoiling activity of DNA gyrase, using inhibition assay of *E. coli* Gyrase enzyme. Studying the dose-response inhibition as shown in (Figure 5), we calculated the 50% inhibition using inhibitor (**2a**) and it was 12.03 µM as compared with the standard antibiotic ciprofloxacin that showed IC_50_ 10.71 µM and with increasing the concentration, the inhibitory effect of the compound (**2a**) increases. The lower IC_50_ value indicates the relative affinity of inhibitor (**2a**) towards gyrase enzyme. Therefore, we postulated that compound (**2a**) was more specific towards DNA gyrase enzyme by inhibiting DNA binding to the enzyme and preventing the supercoiling reactions [40,41]. Further, the DNA gyrase cleavable complex is formed with the incubation of DNA and enzyme in the presence of compound (**2a**) that mediated stabilization of a cleavable complex through a cooperative drug binding process to a desaturated DNA pocket caused by the enzyme. This result is consistent with Otter and his co-works observation [40]. 

**Figure 6:**
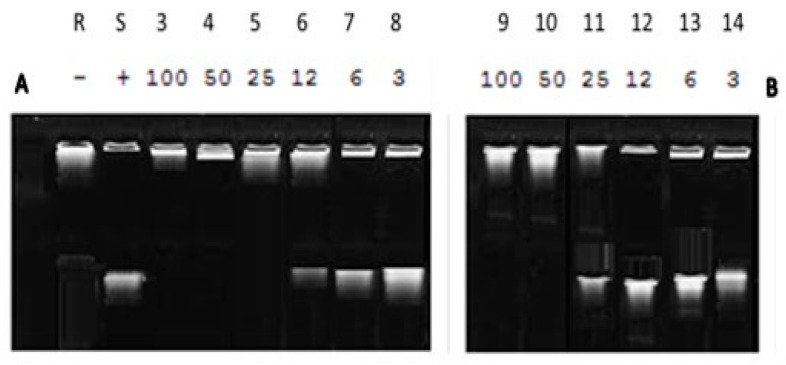
Supercoiling reactions at different concentrations of (**A**) ciprofloxacin (Standard) and (**B**) cyclic diphenylphosphonate **2a**. Lanes 3–14: different concentrations of compound **2a** and ciprofloxacin, respectively (3–14), Lane R: relaxed pNO1, Lane S: supercoiled pNO1 of *E. coli* DNA gyrase.

From the gel electrophoresis, we tested the effect of the inhibitor by showing the difference in the mobility between relaxed and supercoiled DNA. the result showed that the compound (**2a**) has potency in comparison with ciprofloxacin against the enzyme activity explored by inhibiting the DNA gyrase enzyme at different concentrations, where relaxed DNA substrate become completely negatively supercoiled at a lower concentration, and with increasing concentration to 50 µM inhibit supercoiling completely that is also noticed with the addition of ciprofloxacin at 25µM that showed by DNA supercoiling (Figure 6).

### 2.5. Molecular Docking

**Figure 7:**
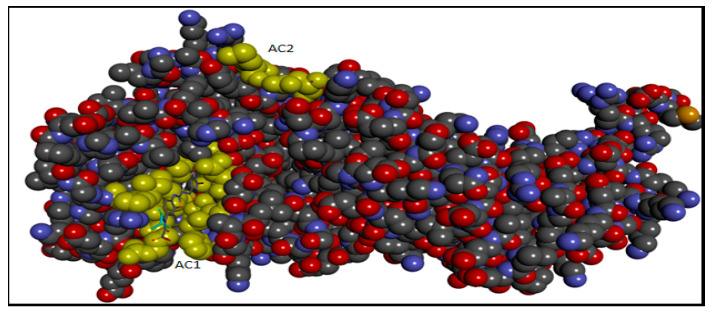
The Crystal structure of DNA gyrase (PDB ID 5L3J) showing two active sites AC1 and AC2.

**Figure 8:**
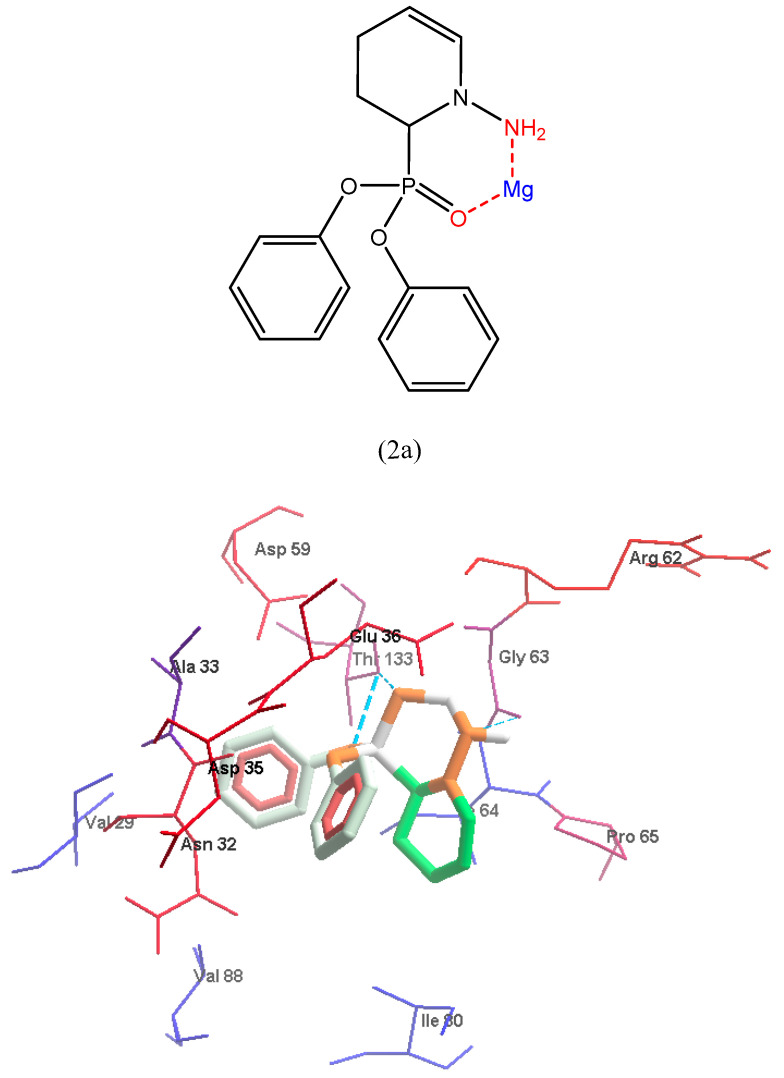
The first view demonstrates hydrogen bonds (blue dash), and hydrophobic interactions between **2a** and gyrase B residues. The hydrophobic parts of **2a** (2 light gray phenyl groups) were enclosed with red residues Asn32 and Asp35; Three hydrogen bonds (blue dash line) interaction one with NH group and Gly33 (NH-Gly63, 3 Å) besides 2 HBs with 2 oxygen atoms of phosphonate groups; Thr133(O-Thr133, 3.3 Å) and (O-Thr133, 2.8 Å). Figure 8 was mapped by Molegro Molecular Viewer, it uses the Kyte-Doolittle scale to rank amino acid hydrophobicity, where the color blue indicates the most hydrophilic, the color white is equal to 0.0, and orange-red color being the most hydrophobic. It also displays the amino acids of gyrase B as thin sticks while compound **2a** atoms are represented as bold sticks.

**Figure 9:**
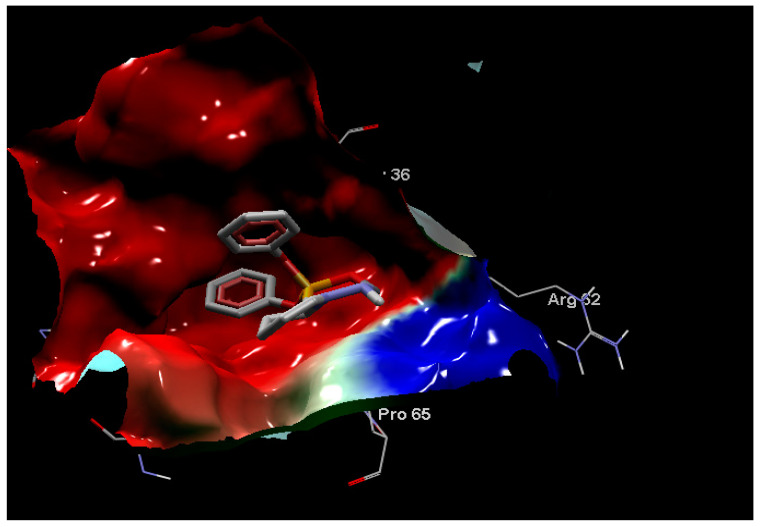
The second view represents the electrostatic interactions of **2a**-gyrase B complex. The second view displays the electrostatic charges, red surface refers to the negative charge while blue surface is the positive charge and the inhibitor **2a** is colored according to the atom type (carbon atom is dark gray, oxygen atoms are red while phosphorus is dark yellow and nitrogen for violet).

A molecular docking study was performed to offer a molecular basis for the potency and selectivity of the here reported compounds. We presume that understanding the interactions between cyclic-diphenylphosphonates and DNA gyrase is necessary for a good and selective antibacterial drug design and development. The inhibitor **2a** is considered the most fitting inhibitor to Active site 1 (AC1) pocket with good binding energy as discussed in the methodology part because of its small size when compared to the other analogs as shown in (Figure 7, Figure 8 and Figure 9).

**Figure 10:**
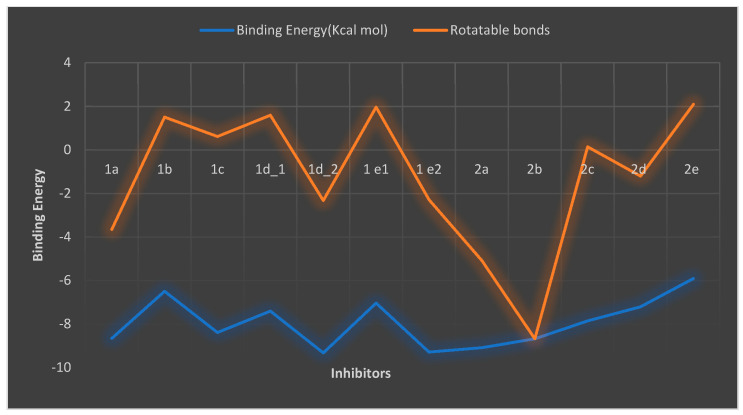
The rotatable bonds effect on the binding energy of gyrase B-inhibitor complex.

The docking results revealed that compound **2a** affects DNA supercoiling through N-terminal domain of gyrase B subunit targets ATP-binding sites that are supposed to compete with ATP for binding to gyrase B, Therefore, we suggest that the mode of action of compound **2a** was similar to the action/mechanism of antibacterial agents as amino-coumarins, novobiocin, and some bacterial toxins such as CcdB and microcin B17 (MccB17), and newly molecule benzothiazole exerting slow inhibition of DNA supercoiling which causing bacterial cell death [42,43,44,45]. Similar to coumarins that competitively inhibit ATP hydrolysis, whereas quinolones, such as ciprofloxacin were stabilizing the covalent gyrase-DNA cleavage complex. CcdB also stabilizes a cleavage complex but only in the presence of ATP [46]. The trapping of the gyrase-DNA cleavage complex by MccB17 is also reported to be ATP-dependent [45]. In addition, the rotatable bonds count has a synergistic effect on binding energy as listed in (Figure 10) and Table 1. A rotatable bond is any single non-ring bond, bound to a non-terminal heavy atom; it detects the conformational entropy change in protein-inhibitor interactions. Rotatable bonds are related to molecular flexibility, which is a good descriptor of oral bioavailability [47]. All the current compounds were bound to gyrase B in AC1 except **1e1** which is bound to Active site 2 (AC2) and different types of interactions were depicted as shown in the supplementary file (Figures S1–S11 cf. Supplementary File). 

From Table 1, compound (**2a**) is the most potent inhibitor while **2b**, **1a,** and **1c** are almost equal in their potency, **1d2** and **1e2** (red color) do not exist in the current experimental condition, they may have occurred at different experimental conditions, **2e** is least potent compound.

## 3. Materials and Methods

### 3.1. Microbiological Screening

#### 3.1.1. Bacterial Strains

Nine clinical pathogenic Multi-Drug Resistance (MDR) isolates namely; *Klebsiella pneumonia, E. coli, and* Methicillin-resistant *Staphylococcus aureus* (*MRSA*) that have been previously identified and their antimicrobial susceptibility assay assessed [4,25,26] were used in our experminet. Bacterial isolates were stored in Brain Heart-Glycerol (Oxoid Ltd., Basingstoke, UK) at −80 °C until use. Bacterial isolates were refreshed by incubating overnight at 37 °C on nutrient broth media (Oxoid, Basingstoke, UK), and then sub-cultured on selective media (MacConkey and Mannitol agar, (Oxoid, Basingstoke, UK). Quality control assessment was carried out using standard reference bacterial strains; *Staphylococcus aureus* ATCC^®^ 25923, and *E.coli* ATCC^®^ 11229 that were obtained from Microbiology Lab’s Unit of National Organization for Drug Control and Research (NODCAR).

#### 3.1.2. Screening of Synthesized Compounds against Tested Isolates Using Agar Well Diffusion Method

Synthesized compounds were prepared for Antibacterial Activity by dissolving in Dimethyl Sulfoxide (DMSO) and then diluted in sterile distilled water to obtain a final concentration of 100 mg/mL. To check the effect of solvent on the growth of microorganisms, DMSO (100 µL) was used as negative control [48]. The antibacterial activity of different compounds was evaluated by agar well diffusion method. Briefly, 100 μL of 0.5 MacFarland bacterial suspensions (1.5 × 10^8^ CFU/mL) was spread on Müller-Hinton agar (Oxoid, Basingstoke, UK). Wells were made on the agar plates using sterile cork borer, then 100 μL of each prepared compound was introduced into appropriately marked wells. The antibacterial activity was evaluated by measuring the diameter of the inhibition zone for each tested organism compared with the negative and positive control after an incubation period of 24 h at 37 °C. DMSO (100 µL) was used as a negative control, and an antibiotic disc of Ciprofloxacin (5 μg) was used as a positive control [48,49].

#### 3.1.3. Quinolone Resistance Mechanism Detection (QRDR): Amplification and Sequencing of *gyrA* in QRDR DNA

QRDR regions of *gyrA* gene of both clinical isolate CIP-resistant *Klebsiella pneumoniae* (Kp8) and CIP-resistant *Escherichia coli* (E17) were amplified by PCR from the chromosomal DNA of quinolone-resistant isolates. A 344-bp region covering the QRDR of *gyrA* was amplified with primers 5′-AAATCTGCCCGTGTCGTTGGT-3′and 5′- GCCATACCTACTGCGATACC-3′ [50]. PCR was performed as recommended, and PCR products were visualized by 1% agarose gel electrophoresis using ethidium bromide [51]. PCR products were purified with high pure PCR product purification kits (QIAquick PCR Purification Kit, Qiagen, Hilden, Germany). Purified PCR products were sequenced using Applied Biosystems 3500 Genetic Analyzer (Hitachi, ThermoFisher, MA, USA) in Colors labs, Egypt. The primers used for sequencing were the same used for amplification. BigDye Terminator v3.1 Cycle Sequencing Kit and 5× Sequencing Buffer (ThermoFisher) were used. 

#### 3.1.4. Impact of Synthesized Compound (**2a**) against DNA Gyrase Activity Using DNA Supercoiling Assay

In vitro, the effect of synthesized compound (**2a**) as a quinolone inhibitor compared with ciprofloxacin (reference standard) at different concentrations was determined using *E. coli* Gyrase Microplate Assay Kit (Inspiralis, Norwich, UK) based on the DNA Supercoiling using DNA gyrase enzyme according to manufacturer’s instructions [52]. The test was carried out in the confirmatory diagnostic unit, Vacsera, Egypt. The inhibition test was carried out using *E. coli* DNA gyrase to determine IC_50_ value. The compound (**2a**) and reference standard were dissolved in DMSO and serially diluted at different concentrations, the assay was carried out in three replicates. Briefly, the reaction was started by rehydrating the wells with 3 × 200 µL wash buffer then zimmobilize 100 µL of 500 nM TFO1 oligo in each well (5 µL of 10 µM TFO1 in 95 µL wash buffer), incubating for 5 min at room temperature. The excess of oligo Wash off with 3 × 200 µL with wash Buffer. 1.5 U of gyrase incubates with 0.75 µg of relaxed plasmid pNO1 in a reaction volume of 30 µL at 37 °C for 30 min in assay buffer. After incubation, we add 100 µL TF buffer to the well and incubate it for a further 30 min at room temperature to allow triplex formation. We remove the unbound plasmid from the well by washing it with 3 × 200 µL TF buffer. The reaction is stained with DNA-detection dye (diluted to 1× with T10 buffer) then we add 200 µL per well and incubate for 10–20 min, then mix and read in a fluorescence plate reader at Ex: 495 nm; Em: 537 nm).

A 10 µL sample was removed from the well for gel assay and was determined using a standard 1% agarose gel assay depending on quantitation of the gel using the intensity of the ethidium fluorescence of the supercoiled DNA band. The IC_50_ was defined as the concentration causing 50% inhibition of the supercoiling reaction. The average IC_50_ values (µM) of the replicate experiments were calculated for the target and reference standard using non-linear regression analysis in GraphPad Prism 7 (GraphPad softwear, San Diego, CA, USA).

### 3.2. Molecular Docking

The molecular docking technique involves receptor preparation, ligand preparations, detection of the active site, and re-establishing a complex of the 3 dimensions (3D) co-crystal structure of receptor and 3D modeled ligand structures.

#### 3.2.1. Preparation of Macromolecules and Ligands

The protein structure was prepared for docking by The UCSF Chimera software [53]. The ligands were drawn and created using Avogadro software [54], Mg^2+^ was the metal-coordinating ligand due to the affinity of cyclic diphenylphosphonate nitrogen donor atoms toward metal ions. About two different chelation modes were produced with Mg^2+^ cation in some compounds. The complexation of ligand monomer with Mg^2+^ was 1:1 molar ratios and minimized by the Avogadro software. Metal chelation of Mg^2+^ with ligand monomer interacted through N3 atom in the imidazole group of the monomer.

#### 3.2.2. Active Site Detection 

The N-terminal fragment of the *E. coli* DNA gyrase B (residues Gly15 to Thr392) was discovered from the X-ray crystal structure 5L3J with resolution 2.83 Å [55], it was used to investigate the binding affinity of the current inhibitors to DNA gyrase B. The crystal structure 5L3J contains two active sites which are AC1 and AC2 where AC1 are: Val43, Asn46, Glu50, Gly77, Ile78, Pro79, Val120, Thr165, and Val167; while AC2 is Gly24 and Gln335, (Figure S12 cf. supplementary file).

Several interactions were shown in (Figure S12 cf. supplementary file), where amide Pi-stacked interaction was confirmed with residue Asn46; while van der Waals interaction exhibited through Glu50, His55, Asp73, Arg76, Gly77, Met 95, and Arg136. Pi-Sigma interaction was exhibited by Thr165 also, Pi-alkyl with Val43, Ala47, Val120, and Val167. 

#### 3.2.3. Molecular Docking Implementation Process

Targeting binding sites with automated docking [56,57] was used to dock ligands to identify the active entities and determine the binding sites in target proteins. Autodock 4.2 [58] was used for docking the current compounds understudy to 5L3J protein. Lamarckian Genetic Algorithm (LGA) was used to determine the globally optimized conformation. LGA for docking was implemented with defined parameters for determining the docking performance. Polar hydrogen atoms were added Kollman charge, atomic solvation parameters, and fragmental volumes were assigned to the protein using Autodock tools. The grid spacing was 0.375 Å for each spacing. Molegro Molecular Viewer packages [59] have been used to visualize 5L3J crystal structure and its binding mode. BIOVIA Discovery Studio 2020 [60] was used to illustrate enzyme-inhibitor interactions.

## 4. Conclusions

Two series of cyclic diphenylphsophonates (**1a**–**e** and **2a**–**e**) were synthesized and evaluated for their in vitro antibacterial activity against ciprofloxacin-resistant *E.coli* and *Klebsiella pneumoniae* clinical isolates. The results of in vitro antibacterial activity in combination with docking study revealed that installation of hydrazine moiety without quinoline and carbon spacer highly contributed to the activity as in compound (**2a**). Further SARs study, demonstrated that the difference in antibacterial activity was a result of the variation of substitutions on the ring nitrogen attached to the chiral carbon atom bearing diphenylphosphonate structural motif. In addition, the experimental and docking studies were compatible with the observed potency of compound (**2a**) as the most potent DNA gyrase inhibitor with an IC_50_ of 12.03 µM. Further variations in substitution patterns may be necessary to obtain more potent and selective DNA-gyrase inhibitors competent in facing the increasing antibacterial resistance.

## Figures and Tables

**Table 1:** Binding energy of the two series gyrase B inhibitors.

Compound	Binding Energy (Kcal mol)	Rotatable Bonds
**1a**	−8.65	5
**1b**	−6.49	8
**1c**	−8.38	9
**1d1**	−7.4	9
**1d2**	−9.32	7
**1e1**	−7.03	9
**1e2**	−9.28	7
**2a**	−9.08	4
**2b**	−8.67	0
**2c**	−7.85	8
**2d**	−7.2	6
**2e**	−5.9	8

## Data Availability

The data used to support the findings of this study are available from the corresponding author upon request.

## Supplementary Materials

The following are available online at https://www.mdpi.com/article/10.3390/antibiotics11010053/s1, Figures S1–S11: display the hydrophobicity interaction of inhibitor for compounds 1 and 2 in AC1 site and their the electrostatic charges, Figure S12:The experimental ligand interaction and distance with the crystal structure 5L3J in AC1. Table S1: Antibacterial activity of cyclic diphenyl phosphonate against tested Gram-positive and Gram-Negative bacteria using agar well diffuion method towards tested two groups of synthetic chemical compounds at concentration 100 mg/mL A: 1 a–e, and B: 2 a–e.
