# Supplementary material for: Experimental and Molecular Docking Studies of Cyclic Diphenyl Phosphonates as DNA Gyrase Inhibitors for Fluoroquinolone-Resistant Pathogens
Source: Antibiotics (Basel). 2022 Jan 1;11(1):53. doi: 10.3390/antibiotics11010053 (PMC8772930; doi:10.3390/antibiotics11010053)
Supplement: Supplementary file 1 [file antibiotics-11-00053-s001.zip › antibiotics-1444135-supplementary.pdf]

antibiotics

Article

Experimental and Molecular Docking Studies of Cyclic Diphenyl Phosphonates as DNA Gyrase Inhibitors for Fluoroquinolone-Resistant Pathogens

Neveen M. Saleh [1,*], Yasmine S. Moemen [2], Sara H. Mohamed [1], Ghady Fathy [3], Abdullah A. S. Ahmed [3], Ahmed A. Al-Ghamdi [4], Sami Ullah [5,6] and Ibrahim El-Tantawy El Sayed [3,*]

1 Department of Microbiology, National Organization for Drug Control and Research, Giza 12553, Egypt; sara_hussein_moh@yahoo.com
2 Clinical Pathology Department, National Liver Institute, Menoufia University, Shebin El-Kom 32511, Egypt; yasmine.moemen@gmail.com
3 Chemistry Department, Faculty of Science, Menoufia University, Shebin El-Kom 32511, Egypt; ghadyfathy96@gmail.com (G.F.); chemist_abdullah_2009@yahoo.com (A.A.S.A.)
4 Department of Physics, Faculty of Science, King Abdulaziz University, Jeddah 21589, Saudi Arabia; agamdi@kau.edu.sa
5 Research Center for Advanced Materials Science (RCAMS), King Khalid University, P.O. Box 9004, Abha, 61413, Saudi Arabia; samiali@kku.edu.sa
6 Department of Chemistry, College of Science, King Khalid University, P.O. Box 9004, Abha 61413, Saudi Arabia; samiali@kku.edu.sa
* Correspondence: salehneveen@yahoo.com (N.M.S.); ibrahimtantawy@yahoo.co.uk (I.E.-T.E.S.)

**Com. (100mg/ml)**	**Bacterial Species /Inhibition Zones mm ± SD***										
	**Gram-Negative Bacteria**								**Gram-Positive Bacteria**		
	CIP-resistant *E. coli*				CIP-resistant *Klebsiella pneumonia*				*E.coli* ATCC 11229	*Staph. aureus* ATCC 25923	*MRSA Staph. aureus* SA4
	E15	E16	E17	E13	Kp1	Kp8	Kp9	Kp5			
1a	1.2 ± 0	1.2 ± 0	1.3 ± 0.05	0	0	0	0	1.2 ± 0	1.85± 0.05	0	0
1b	3.1 ± 0.1	0	0	2.3 ± 0.1	0	0	0	0	2.2± 0.05	0	0
1c	1.5 ± 0	1.3 ± 0	1.5 ± 0	1.7 ± 0	1.5 ± 0	1.05 ± 0.05	1.25 ± 0.05	0	2.0± 0.05	0	0
1d	0	0	0	0	0	0	0	1.03 ± 0.05	1.5± 0.05	1.05± 0.0	0
1e	0	0	0	0	0	0	0	1.15 ± 0.05	1.8± 0.05	1.15± 0.0	1.8± 0.05
2a	2 ± 0	2 ± 0	3 ± 0	2.2 ± 0	1.7 ± 0	2.7 ± 0	1.9 ± 0	3.6 ± 0	2.95± 0.05	2.5± 0	1.85± 0
2b	0	0	0	0	2.2 ± 0	1.15 ± 0.05	1.9 ± 0.1	0	1.85± 0.05	1.85± 0	1.35± 0.05
2c	0	0	0	0	2 ± 0	1.55 ± 0.05	2 ± 0	0	2.2± 0.05	0	0
2d	0	0	0	0	0	0	0	1.03 ± 0.05	1.5± 0.05	1.05± 0.0	0
2e	0	0	0	0	0	0	0	1.15 ± 0.05	1.8± 0.05	1.15± 0.0	1.8± 0.05
CIP [1]	0	0	0	0	0	0	0	0	20	22.5	22.5

[1] CIP: Ciprofloxacin Reference standard.

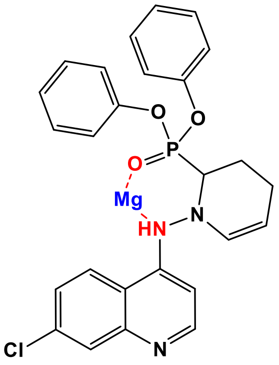

(1a)

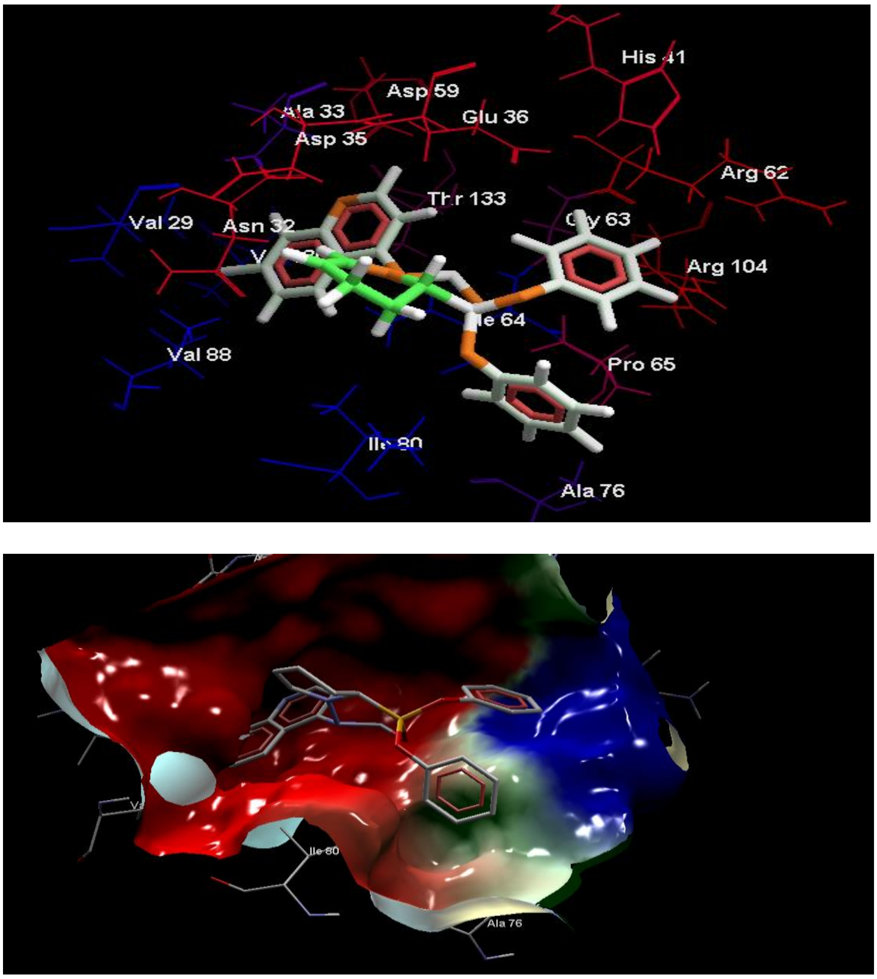

Figure S1. the first view display the hydrophobicity interaction of inhibitor 1a in AC1 site. The first view displays hydrophobic interaction between 1a and gyrase B which mapped by Molegro Molecular Viewer, it uses the Kyte-Doolittle scale to rank amino acid hydrophobicity, where the color blue in-dicating the most hydrophilic, the color white equal 0.0 and orange-red color being the most hydrophobic. It also displays the amino acids of gyrase B as thin sticks while compound 1a atoms are represented as bold sticks.The second view represents the electrostatic interactions of 1a-gyrase B complex. The second view display the electrostatic charges, red surface refers to negative charge while blue surface is positive charge and the inhibitor 1a colored according to the atom type (carbon atom is dark gray, oxygen atoms are red while phosphorus is dark yellow and nitrogen for violet).

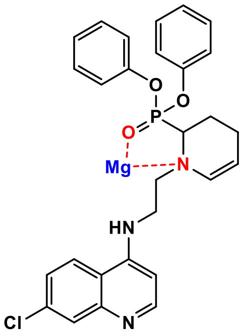

(1b)

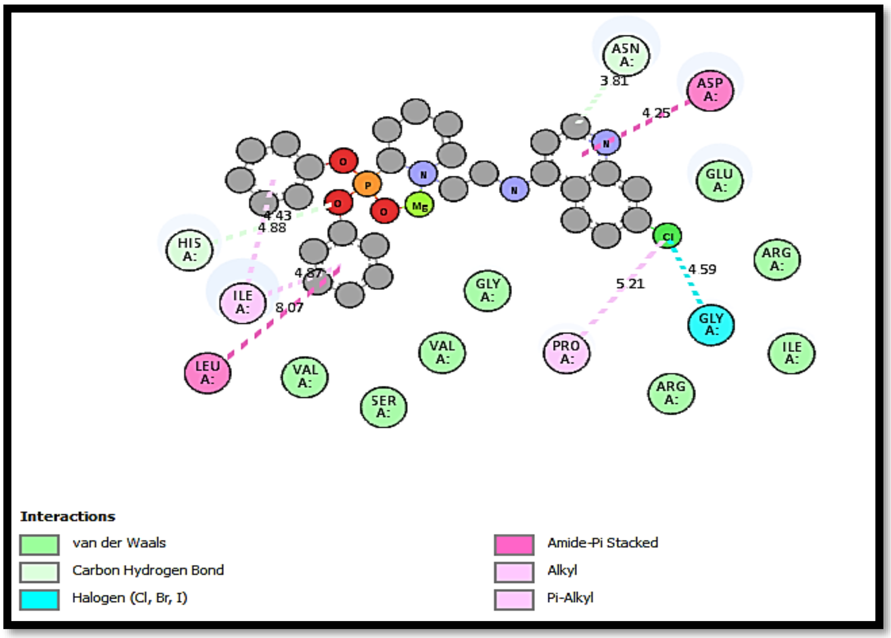

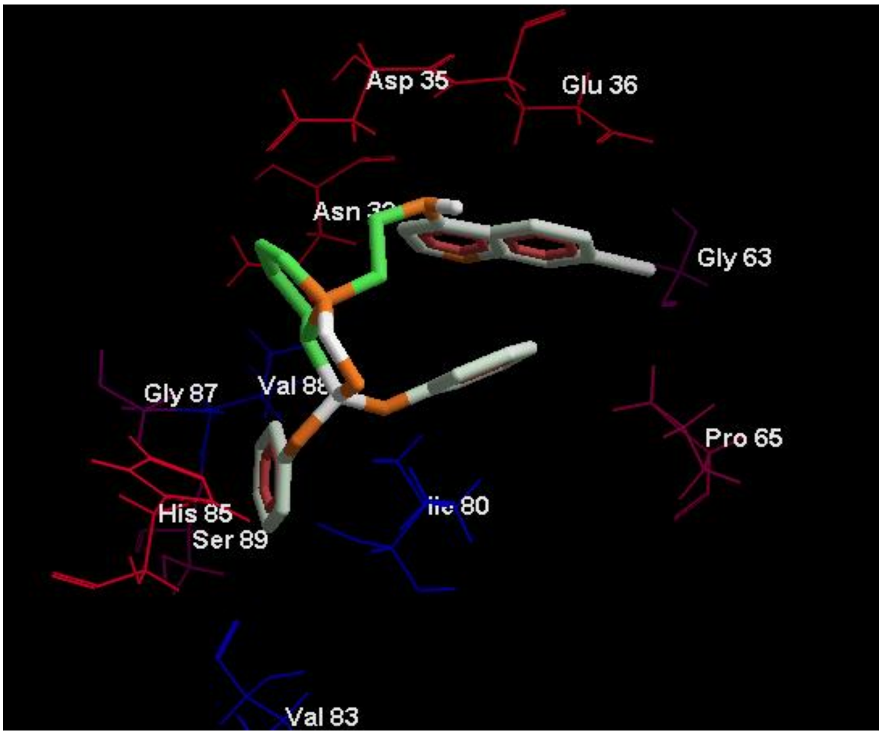

Figure S2. the first view represents different interactions for **1b** in AC1 site. the second view displays the hydrophobicity interaction of inhibitor **1b** in AC1 site. The second view displays hydrophobic

interaction between **1b** and gyrase B which mapped by Molegro Molecular Viewer, it uses the Kyte-Doolittle scale to rank amino acid hydrophobicity, where the color blue indicating the most hydrophilic, the color white equal 0.0 and orange-red color being the most hydrophobic. It also displays the amino acids of gyrase B as thin sticks while compound 1b atoms are represented as bold sticks.

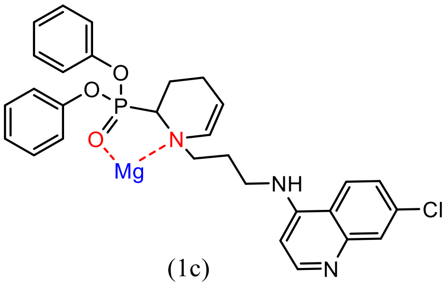

(1c)

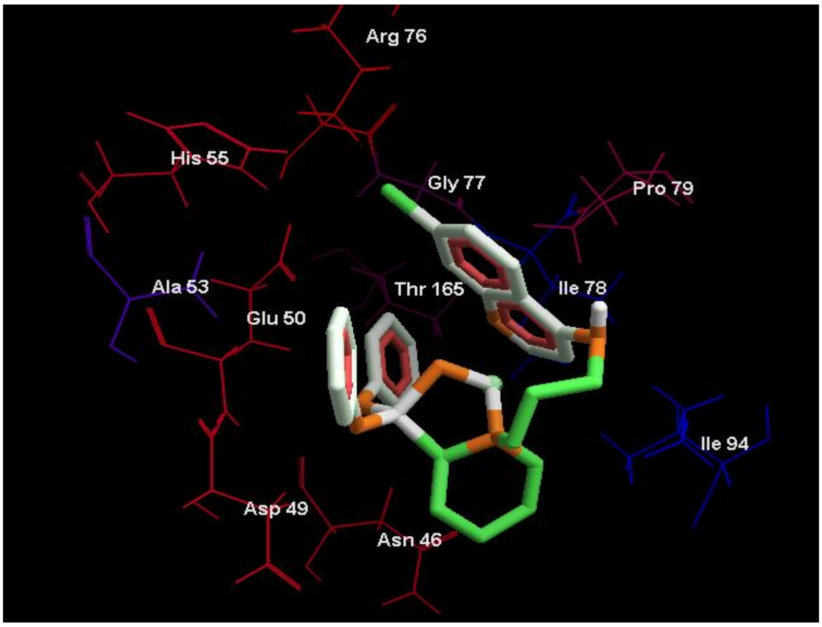

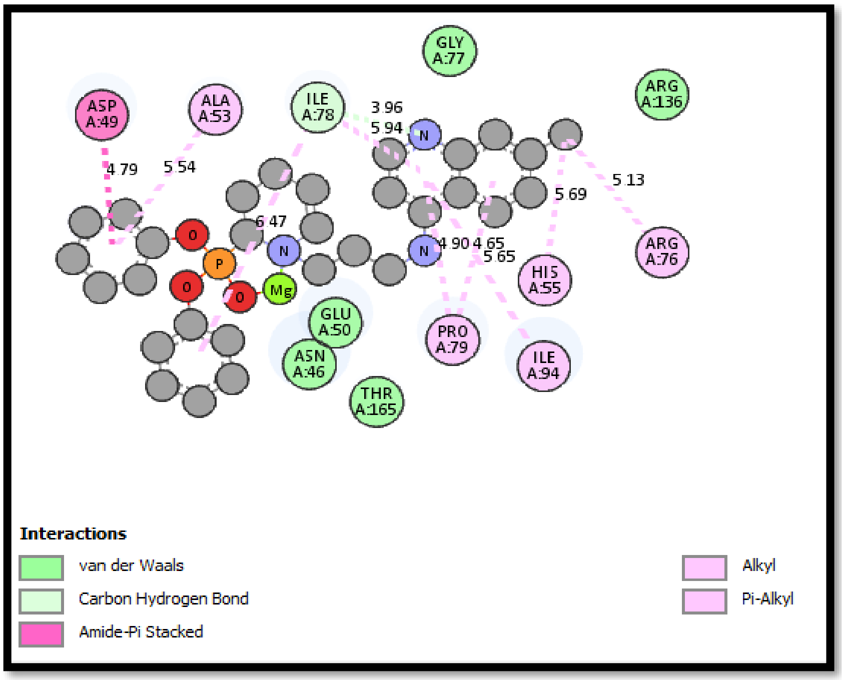

Figure S3. The first view displays hydrophobicity interaction of inhibitor **1c** in AC1 site The first view displays hydrophobic interaction between **1c** and gyrase B which mapped by Molegro

Molecular Viewer, it uses the Kyte-Doolittle scale to rank amino acid hydrophobicity, where the color blue indicating the most hydrophilic, the color white equal 0.0 and orange-red color being the most hydrophobic. It also displays theamino acids of gyrase B as thin sticks while compound **1c** atoms are represented as bold sticks. the second view exhibit different interactions for **1c** in AC1 site.

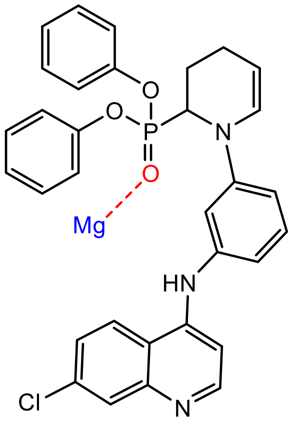

(1d1)

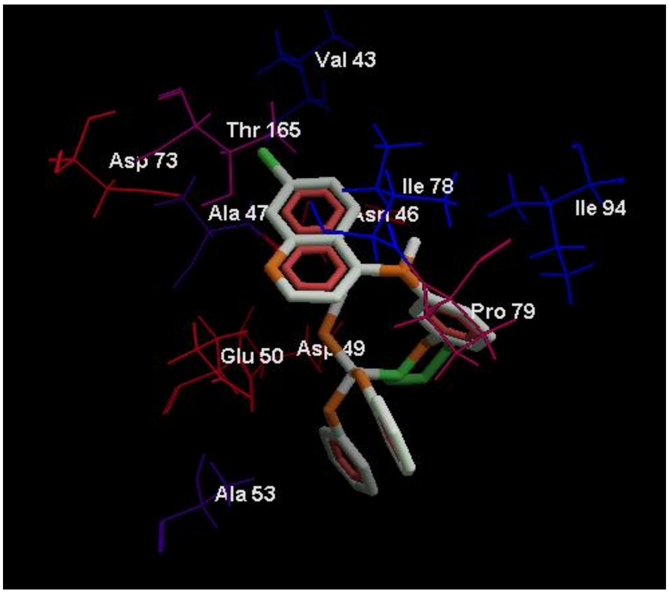

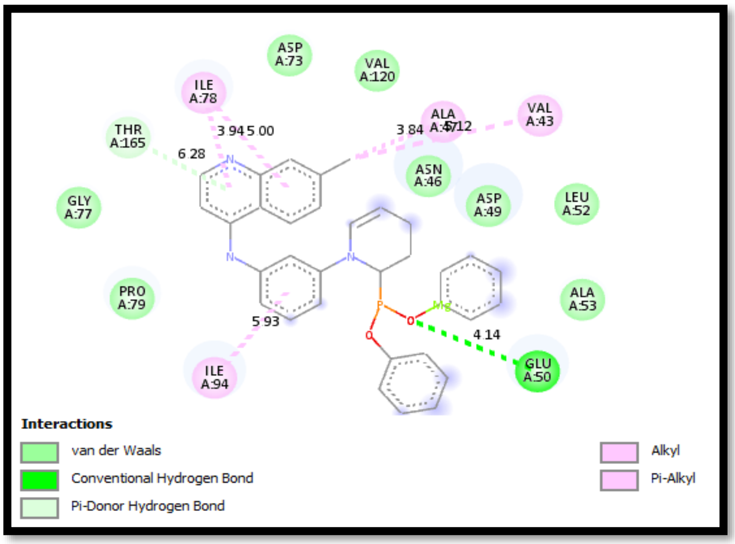

Figure S4. The first view displays hydrophobicity interaction of inhibitor 1d1 in AC1 site. This view displays hydrophobic interaction between **1d1** and gyrase B which mapped by Molegro Molecular Viewer, it uses the Kyte-Doolittle scale to rank amino acid hydrophobicity, where the color blue indicating the most hydrophilic, the color white equal 0.0 and orange-red color being the most hydrophobic. It also displays the amino acids of gyrase B as thin sticks while compound 1d1 atoms are represented as bold sticks. the second view exhibit different interactions for **1d1** in AC1 site. the second view exhibit different interactions for **1d1** in AC1 site.

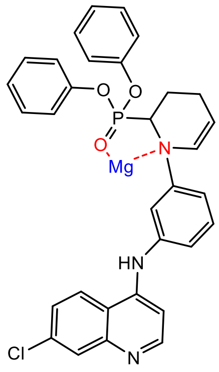

(1d2)

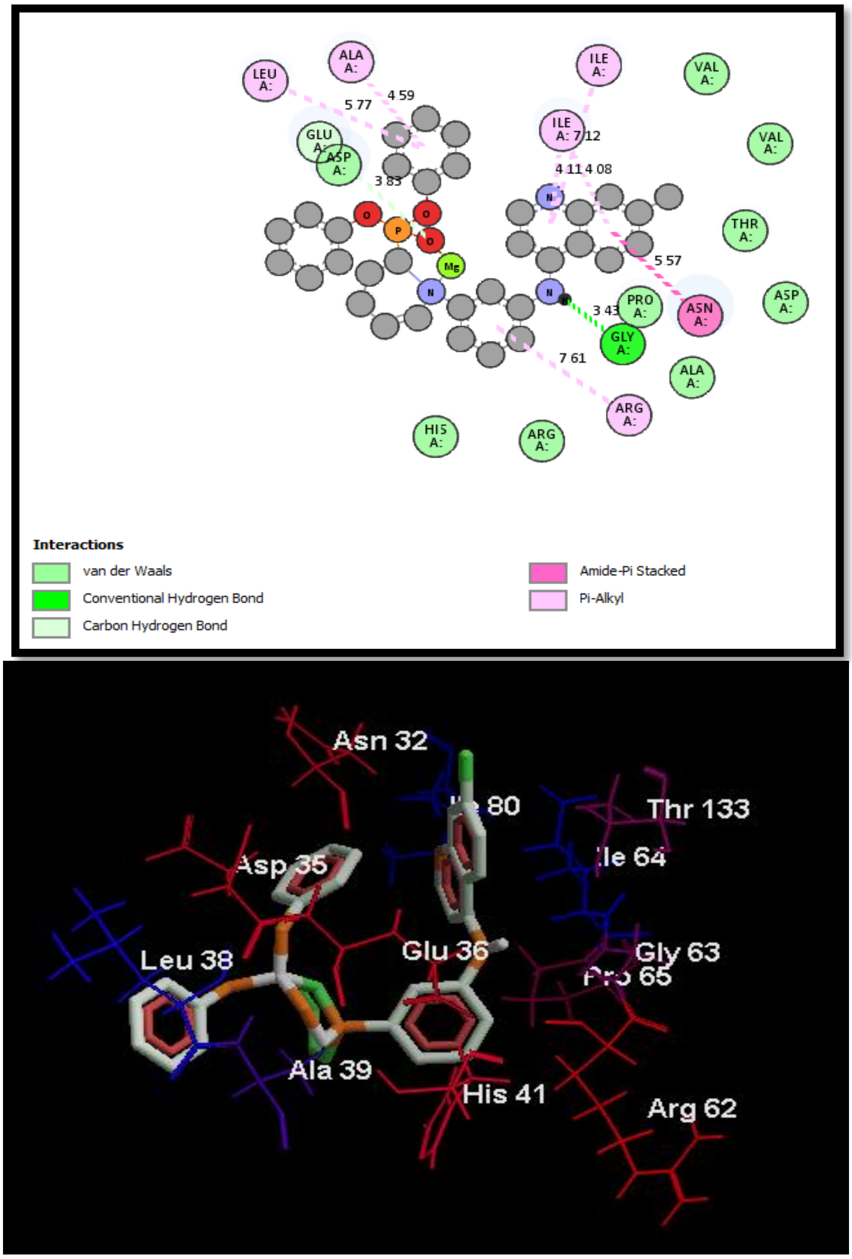

Figure S5. the first view represents different interactions for **1e1** in AC2 site. The first view displays hydrophobic interaction between **1e1** and gyrase B which mapped by Molegro Molecular Viewer, it uses the Kyte-Doolittle scale to rank amino acid hydrophobicity, where the Colour blue indicating the most hydrophilic, the color white equal 0.0 and orange-red color being the most hydrophobic. It also displays the amino acids of gyrase B as thin sticks while compound **1e1** atoms are represented as bold sticks. the second view displays the hydrophobicity interaction of inhibitor **1e1** in AC2.

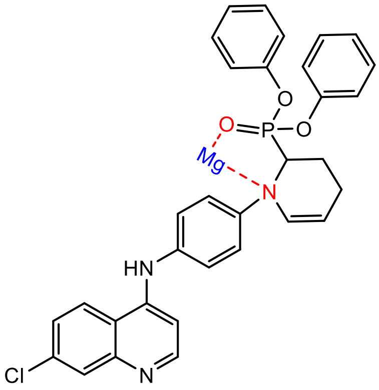

(1e2)

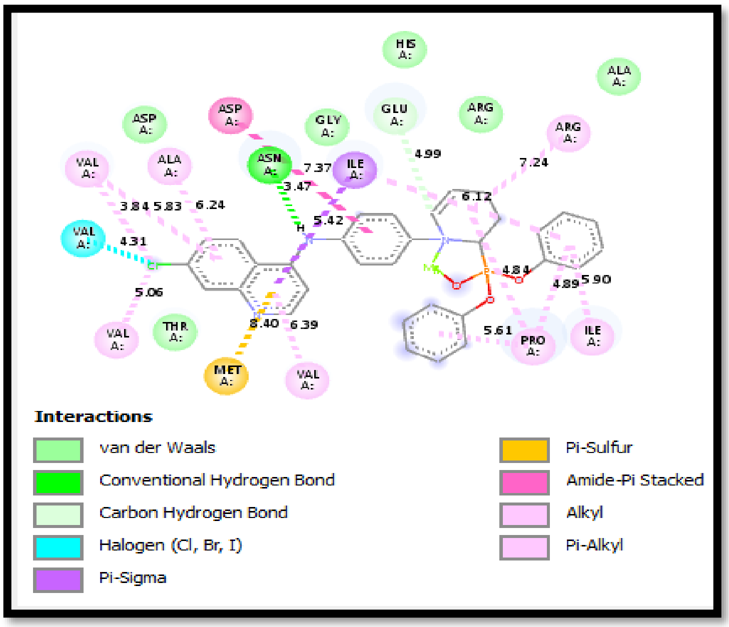

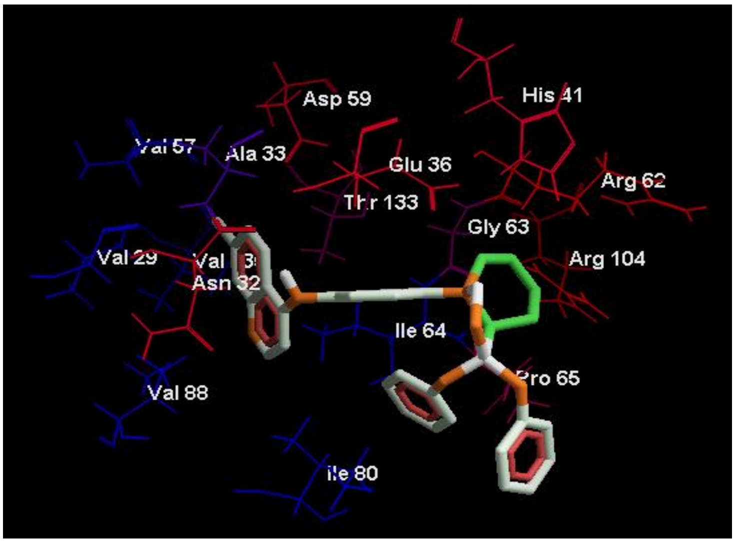

Figure S7. the first view represents different interactions for **1e2** in AC2 site. The first view displays hydrophobic interaction between **1e2** and gyrase B which mapped by Molegro Molecular Viewer, it uses the Kyte-Doolittle scale to rank amino acid hydrophobicity, where the color blue indicating the most hydrophilic, the color white equal 0.0 and orange red color being the most hydrophobic. It also displays the amino acids of gyrase B as thin sticks while compound **1e2** atoms are represented as bold sticks. the second view displays the hydrophobicity interaction of inhibitor **1e2** in AC2.

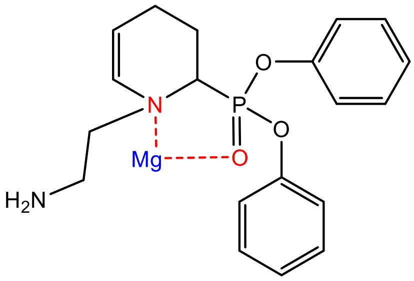

(2b)

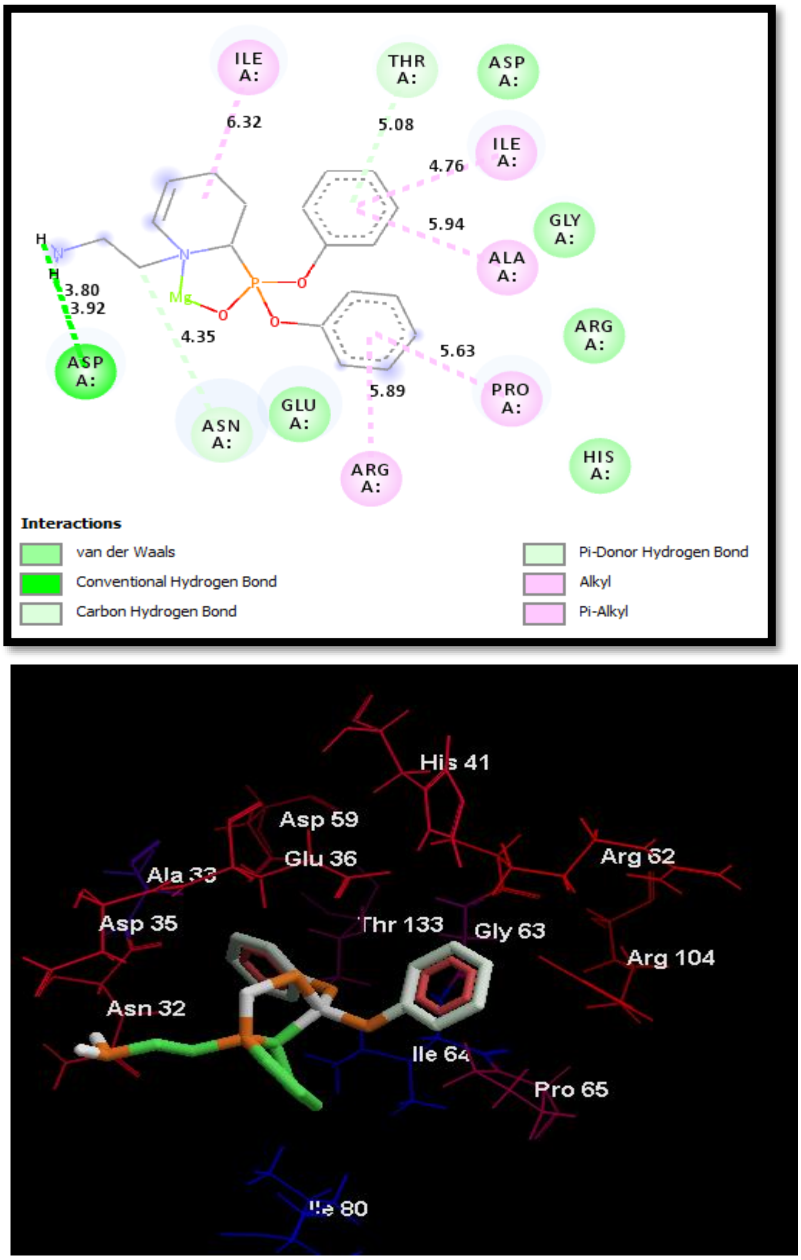

Figure S8. the first view represents different interactions for **2b** in AC1 site The first view displays hydrophobic interaction between **2b** and gyrase B which mapped by Molegro Molecular Viewer, it uses the Kyte-Doolittle scale to rank amino acid hydrophobicity, where the color blue indicating the most hydrophilic, the color white equal 0.0 and orange-red color being the most hydrophobic. It also displays the amino acids of gyrase B as thin sticks while compound **2b** atoms are represented as bold sticks. the second view displays the hydrophobicity interaction of inhibitor **2b** in AC1.

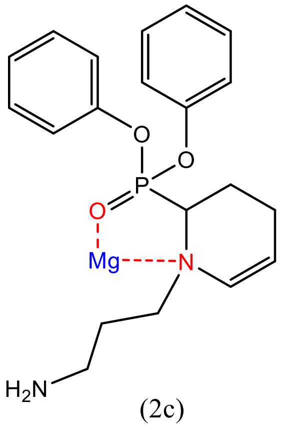

(2c)

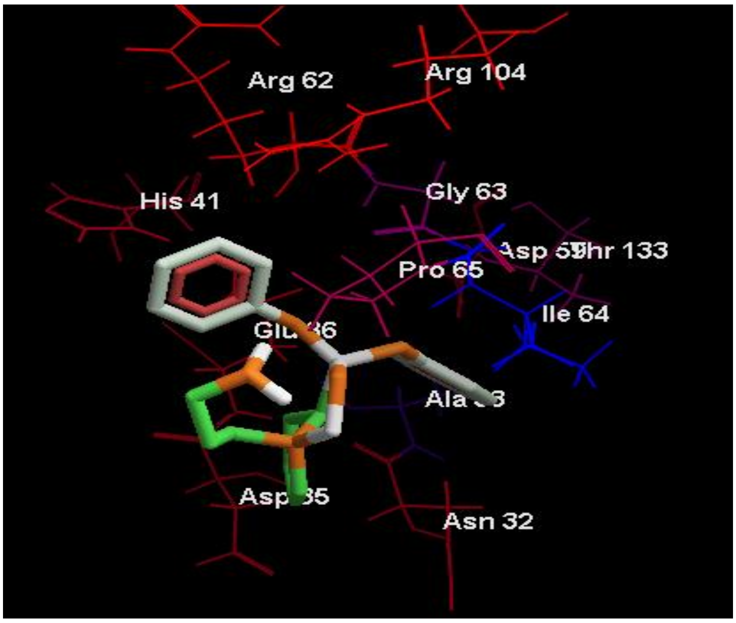

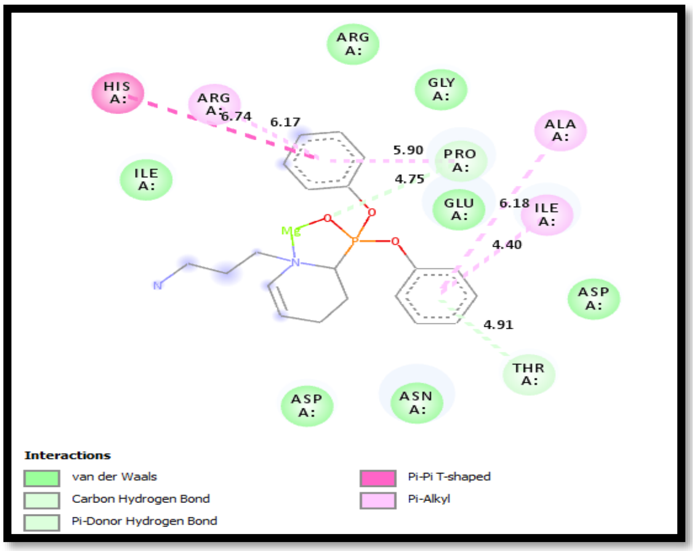

Figure S9. The first view displays hydrophobicity interaction of inhibitor **2c** in AC1 site. The first view displays hydrophobic interaction between **2c** and gyrase B which mapped by Molegro Molecular Viewer, it uses the Kyte-Doolittle scale to rank amino acid hydrophobicity, where the color blue indicating the most hydrophilic, the color white equal 0.0 and orange-red color being the most hydrophobic. It also displays the amino acids of gyrase B as thin sticks while compound **2c** atoms are represented as bold sticks. the second view exhibit different interactions for **2c** in AC1 site.

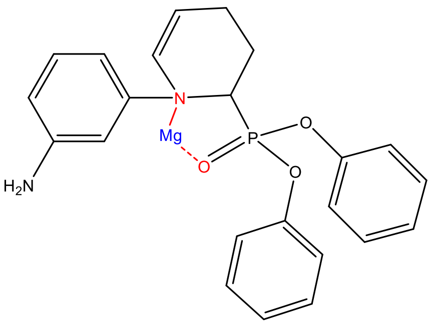

(2d)

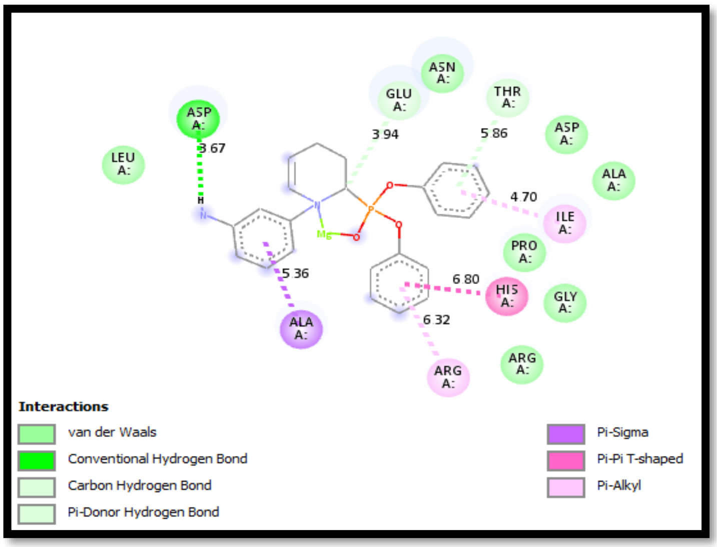

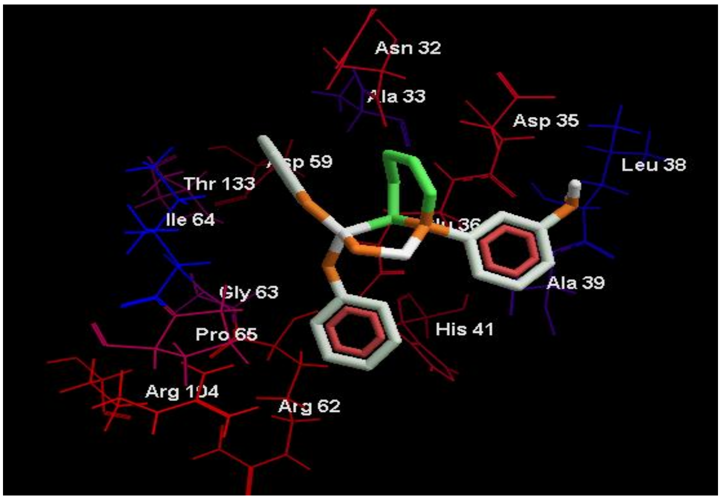

Figure S10. The first view displays hydrophobicity interaction of inhibitor **2d** in AC1 site. the second view exhibit different interactions for **2d** in AC1 site. The second view displays hydrophobic interaction between **2d** and gyrase B which mapped by Molegro Molecular Viewer, it uses the Kyte-Doolittle scale to rank amino acid hydrophobicity, where the color blue indicating the most hydrophilic, the color white equal 0.0 and orange-red color being the most hydrophobic. It also displays the amino acids of gyrase B as thin sticks while compound **2d** atoms are represented as bold sticks.

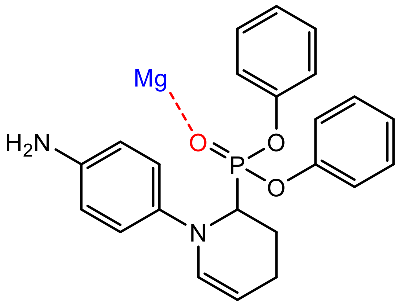

(2e)

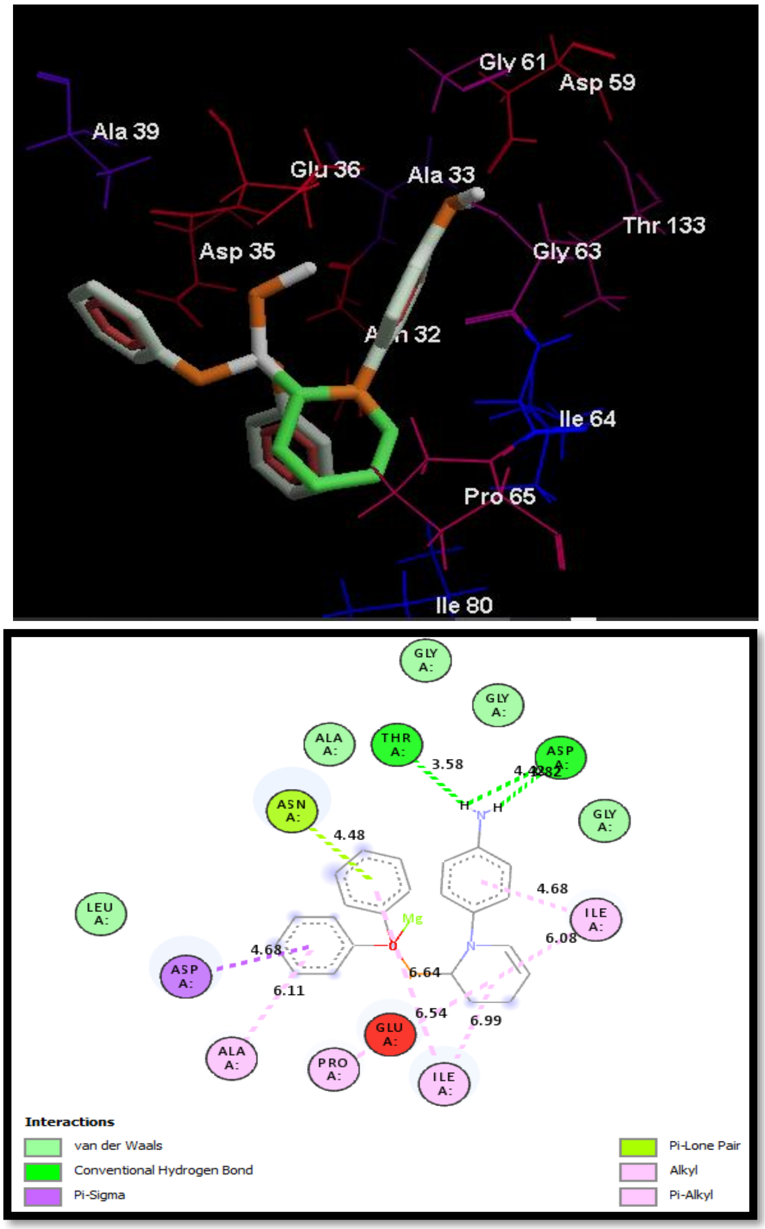

Figure S11. The first view displays hydrophobicity interaction of inhibitor **2e** in AC1 site. The first view displays hydrophobic interaction between **2e** and gyrase B which mapped by Molegro Molecular Viewer, it uses the Kyte-Doolittle scale to rank amino acid hydrophobicity, where the color blue indicating the most hydrophilic, the color white equal 0.0 and orange-red color being the most hydrophobic. It also displays the amino acids of gyrase B as thin sticks while compound **2e** atoms are represented as bold sticks. the second view exhibit different interactions for 2e in AC1 site.

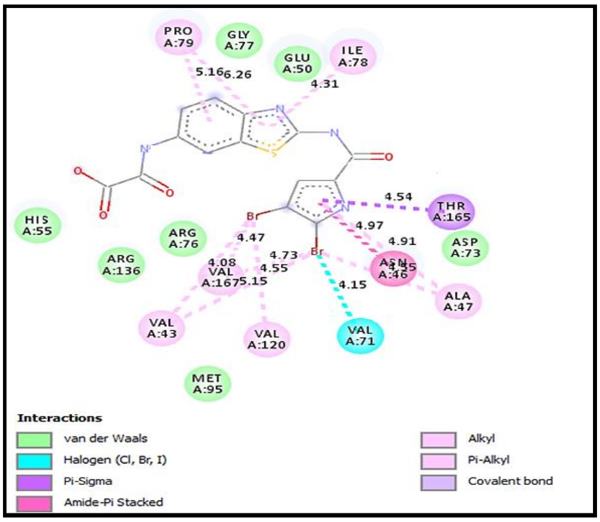

Figure S12. The experimental ligand interaction and distance with the crystal structure **5L3J** in AC1.
